# Developmental Analysis of the GATA Factor *HANABA TARANU* Mutants in *Medicago truncatula* Reveals Their Roles in Nodule Formation

**DOI:** 10.3389/fpls.2021.616776

**Published:** 2021-04-29

**Authors:** Yiteng Xu, Hongfeng Wang, Zhichao Lu, Lizhu Wen, Zhiqun Gu, Xue Zhang, Guangle Yu, Hailong Wang, Chuanen Zhou, Lu Han

**Affiliations:** ^1^The Key Laboratory of Plant Development and Environmental Adaptation Biology, Ministry of Education, School of Life Sciences, Shandong University, Qingdao, China; ^2^State Key Laboratory of Microbial Technology, Institute of Microbial Technology, Helmholtz International Lab for Anti-infectives, Shandong University-Helmholtz Institute of Biotechnology, Shandong University, Qingdao, China

**Keywords:** *Medicago truncatula*, HANABA TARANU, expression analysis, nodule development, NCR genes, Tnt1 mutants

## Abstract

Formation of nodules on legume roots results from symbiosis with rhizobial bacteria. Here, we identified two GATA transcription factors, *MtHAN1* and *MtHAN2*, in *Medicago truncatula*, which are the homologs of *HANABA TARANU* (*HAN*) and *HANABA TARANU LIKE* in *Arabidopsis thaliana*. Our analysis revealed that *MtHAN1* and *MtHAN2* are expressed in roots and shoots including the root tip and nodule apex. We further show that *MtHAN1* and *MtHAN2* localize to the nucleus where they interact and that single and double loss-of-function mutants of *MtHAN1* and *MtHAN2* did not show any obvious phenotype in flower development, suggesting their role is different than their closest Arabidopsis homologues. Investigation of their symbiotic phenotypes revealed that the *mthan1 mthan2* double mutant develop twice as many nodules as wild type, revealing a novel biological role for GATA transcription factors. We found that *HAN1/2* transcript levels respond to nitrate treatment like their Arabidopsis counterparts. Global gene transcriptional analysis by RNA sequencing revealed different expression genes enriched for several pathways important for nodule development including flavonoid biosynthesis and phytohormones. In addition, further studies suggest that *MtHAN1* and *MtHAN2* are required for the expression of several *nodule-specific cysteine-rich* genes, which they may activate directly, and many peptidase and peptidase inhibitor genes. This work expands our knowledge of the functions of *MtHANs* in plants by revealing an unexpected role in legume nodulation.

## Introduction

Legumes have the ability to survive in nitrogen-limited soils by forming root nodules that are colonized by nitrogen-fixing symbiotic bacteria. The formation of nodules requires a complex communication between bacteria and the host plants (Ferguson et al., 2010). The symbiosis begins with exudation secondary metabolites called flavonoids from legume roots which triggers Nod factor production by the rhizobia (Liu and Murray, 2016). The bacterial Nod factors are then perceived by the plant to initiate nodule formation, which forms from differentiated root cortex or pericycle (Via et al., 2016; Buhian and Bensmihen, 2018). The nodules in *Medicago truncatula* consist of several domains, including a persistent apical meristem (zone I), a 10- to 15-cell layer wide infection zone (zone II), a two- to three-cell layer wide interzone (zone II–III), and a growing nitrogen-fixing zone (zone III) (Farkas et al., 2014). Legume nodules initiate from cortical cells which form the nodule primordium. The nodule meristem is formed at the apex of the primordia (Timmers et al., 1999; Stougaard, 2001; Limpens and Bisseling, 2003). Many genes involved in nodulation and symbiotic nitrogen fixation have been isolated in *M. truncatula* (Roy et al., 2020), however, many regulators remain to be discovered due to functional redundancy.

Previous studies demonstrate the expression of several root meristem regulators in the nodule meristem, revealing a relationship between nodulation and lateral root development (Hirsch et al., 1997; Mathesius et al., 2000; de Billy et al., 2001; Bright et al., 2005; Desbrosses and Stougaard, 2011). *MtPLETHORA3* (*MtPLT3*) and *MtPLT4* are expressed not only in the central part of nodule meristem, but also in the root meristem (Schiessl et al., 2019). Knock-down of *MtPLT* genes results in a decrease of nodule number in *M. truncatula* (Franssen et al., 2015). Moreover, *ASL18*/*LBD16a*, a lateral root developmental element, functions in the downstream of *NIN* to drive nodule symbiosis in *Lotus japonicus* (Soyano et al., 2019). These studies indicate that root developmental programs are involved in nodule formation.

Nodule-specific cysteine-rich (NCR) peptides are small legume-specific peptides produced in rhizobium-infected cells, which have multiple functions in nodule development. In *M. truncatula*, about 600 genes code for NCR peptides (Guefrachi et al., 2014). Most *NCR* genes are expressed specifically in nodules, and none of them are induced by Nod factors during symbiosis (Guefrachi et al., 2014). The cationic NCR peptides have antibacterial activity, the application of which *in vitro* increases permeability of bacterial membranes leading to cell death (Tiricz et al., 2013).

Phytohormone signaling pathways are also important for the root nodule symbiosis (Liu et al., 2018). Activation of cytokinin signaling and the level of cytokinin is important for nodule formation and development. A loss-of-function mutation in the cytokinin receptor *cre1* exhibited abundant infection-thread formation, but failed to initiate cortical cell division in *M. truncatula* (Murray et al., 2007). Auxin is also required for both infection thread formation and nodule development (Breakspear et al., 2014) which is regulated by the auxin influx carrier *MtLAX2* (de Billy et al., 2001; Roy et al., 2017). Furthermore, ethylene, gibberellic acid (GAs) and abscisic acid (ABA) play negative roles in nodule formation. The ethylene-insensitive mutant *mtskl* (ortholog of Arabidopsis *ein2*) shows an increased number of infection threads and nodules (Penmetsa and Cook, 1997; Penmetsa et al., 2008). Exogenous application of GA3 inhibits NF-induced root hair deformation, infection thread formation and nodule development in *L. japonicus* and *M. truncatula* (Maekawa et al., 2009; Fonouni-Farde et al., 2016; Jin et al., 2016). Recent work shows that *della* triple mutant exhibits a strong impairment in infection thread formation and nodule development in *M. truncatula* (Jin et al., 2016). Treatment with abamine, a specific ABA biosynthesis inhibitor, can increase nodule number in *L. japonicus* (Suzuki et al., 2004), and exogenous application of ABA inhibits rhizobial infection and nodulation in many legume species (Suzuki et al., 2004; Ding et al., 2008).

GATA factors are transcription regulators that bind to the consensus sequence W-GATA-R [W, thymidine (T) or an adenosine (A); R, guanidine (G) or adenosine (A)] in DNA (Lowry and Atchley, 2000). All GATA transcription factors of Arabidopsis have a type IV zinc finger with the consensus C–X_2_–C–X_17–20_–C–X_2_–C (C, cysteine; X, any residue) followed by a highly basic amino acid stretch (Reyes et al., 2004). There are 29 GATA factors in the Arabidopsis genome, that are divided into four groups depending on sequence conservation, protein domains and gene structure (Reyes et al., 2004). Functional studies have identified the effects of GATA factors in a range of processes including cell elongation (Nishii et al., 2000; Shikata et al., 2004), floral meristem and shoot apical meristem development (Zhao et al., 2004), and seed germination (Liu et al., 2005). One that has been well studied is *HANABA TARANU* (*HAN*). *HAN* participates in many aspects of plant development. In the plant embryo, *HAN* is needed to position the inductive proembryo boundary. Mutation of *HAN* results in the apical redistribution of auxin as early as the eighth cell stage. Developmental defects of *han* are obvious by the 16th cell stage, when the tangential division often fails, and misaligned oblique division is usually observed in the upper tier (Nawy et al., 2010). In the reproductive stage, *HAN* plays roles in establishing boundaries and controlling WUS-expressing cells in shoots and flowers. The *han* mutants in Arabidopsis have flower developmental defects in all four whorls, including fused sepals, reduced numbers of petals and stamens and unfused carpels. The flowers of weak *han* mutant have two to four sepals, one to two petals, four or five stamens, and two asymmetric carpels (Zhao et al., 2004). The genetic combination *han* and *clavata* (*clv*) mutations results in highly fasciated SAMs (Zhao et al., 2004). As a boundary-expressing gene, *HAN* regulates flower development through communicating with the *PINHEAD* (*PNH*), *JAGGED* (*JAG*), *BLADE-ON-PETIOLE 2* (*BOP2*), and *CYTOKININ OXIDASE 3* (*CKX3*) (Ding et al., 2015).

In this study, we identified two members of GATA family in *M. truncatula*, *MtHAN1* and *MtHAN2*, which are homologs of *HAN* and *HANABA TARANU LIKE* (*HANL*). We found that the expression of *MtHAN1* and *MtHAN2* are expressed in nodules and were nitrate regulated. Further genetic study showed that the *mthan1 mthan2* double mutant developed more nodules than wild type. RNA-seq data showed that the DEGs in the *mthan* mutant included *NCRs*, peptidases and peptidase inhibitors and gene involved in multiple pathways including flavonoid biosynthesis and hormone signal transduction.

## Materials and Methods

### Plant Materials and Growth Conditions

*Medicago truncatula* ecotype R108 was used in this study. The *mthan1-1* (NF13128), *mthan1-2* (NF4035), *mthan2-1* (NF4633), and *mthan2-2* (NF5115) mutant lines were identified from a *M. truncatula Tnt1* retrotransposon-tagged mutant collection (Tadege et al., 2008). The plants were cultivated in a growth chamber at 22°C day/20°C night, with a photoperiod of 16-h-day/8-h-night, and relative humidity was 70–80%. *Nicotiana benthamiana* used for BiFC experiments was grown under long-day conditions at 22°C day/20°C night.

### Phylogenetic Analysis

Alignment of multiple protein sequences was performed using online CLUSTALW^1^. The neighbor-joining phylogenetic tree was constructed using the MEGA 4.1 software suite^2^. The most parsimonious trees with bootstrap values from 1,000 trials were shown.

### Plasmids Construction and Plant Transformation

For constructing overexpression vectors, the full CDS of *MtHAN1* and *MtHAN2* were PCR amplified from wild type *M. truncatula* and cloned into the pENTR/D-TOPO cloning vector (Invitrogen), then transferred into the pEarleyGate103 vector by attL × attR recombination reactions (Invitrogen) (Earley et al., 2006). For analysis of gene expression patterns, the pBGWFS7 vector containing 2595bp and 2191bp promotors fragments of *MtHAN1* and *MtHAN2*, respectively, were constructed (Karimi et al., 2002). For stable transformation, binary vector constructs were transformed into ecotype R108 using the disarmed *Agrobacterium tumefaciens* strain EHA105.

### Subcellular Localization of *MtHANs*

For subcellular localization of *MtHANs*, the *A. tumefaciens* EHA105 strain harboring the plasmids pEarleyGate 103-*MtHAN1* and pEarleyGate 103-*MtHAN2* were infiltrated into epidermal cells of tobacco. 48 h later, fluorescence signals were examined using an LSM 880 (Zeiss) confocal laser scanning microscope.

### Yeast Two-Hybrid Assays and BiFC Assay

To test physical interaction between *MtHAN1* and *MtHAN2*, LR interactions were made for pENTR-*MtHAN1* with pDEST22 and pENTR-*MtHAN2* with pDEST32 (Invitrogen) to generate bait and prey plasmids construct. The yeast strain MAV203 was used for transformation of bait and prey plasmids. Yeast transformants were selected on synthetic minimal double dropout medium deficient in Trp and Leu (Clontech). For protein interaction tests, medium supplemented with SD-Leu-Trp-His (Clontech) and 1 mM 3-amino-1, 2, 4 triazole (Sigma) was used.

BiFC assays were conducted as described (Ou et al., 2011), and some modifications were made. LR reactions were used to transfer *MtHAN1* and *MtHAN2* into pEARLEY201-YN, pEARLEY202-YC, respectively, and then the plasmids were transformed into *Agrobacterium* EHA105. To test interactions, pEARLEY201-YN-*MtHAN1* and pEARLEY202-YC-*MtHAN2* were coinfiltrated into 4-weeks-old *N. benthamiana* leaves. After injection, *N. benthamiana* was incubated in darkness for 24 h and then 36 h in light. After that, the leaves were dissected for observation using a LSM 880 confocal laser scanning microscope (Zeiss) for capture of fluorescent images. The 488-nm line of an argon laser was chosen for excitation yellow fluorescent protein (YFP).

### β-Glucuronidase Staining, X-Gal Staining and Microscopy

For β-Glucuronidase (GUS) staining analysis, nodules in different developmental stages were used. GUS activity was histochemically detected as previously described (Wang et al., 2019). The roots and nodules stained with GUS solution were fixed in 3% glutaraldehyde in a phosphate buffer and then dehydrated and embedded in wax. Then the samples were sectioned using a RM 2255 microtome (Leica). For X-gal staining, LacZ activity of nodules was assayed as previously described (Wang et al., 2018), and the sample were embedded in 5% (w/v) agarose, and 50-μm sections were made using a VT1200S vibratome (Leica).

### Phenotypic Analysis

For root morphology analysis, mutant and wild type plants were transferred to MS plates after germination. The length of primary roots was measured on the seventh day.

### Nitrate Treatment

Two-weeks-old seedlings growing in plastic tray were supplied with a solution containing 20 mM KNO_3_ for induction or 20 mM KCl as a control. Leaves and petioles were collected at 0, 2, 4, and 6 h after treatment and total RNA was extracted.

### Nodule Induction

For rhizobial inoculation, seedlings of wild type, *mthan1, mthan2, mthan1 mthan2*, *35S:MtHAN1*, and *35S:MtHAN2* were transferred into a plastic tray filled with perlite/sand (in a 3:1 ratio). Plants growing in the chamber were watered with nutrient solution without nitrate every 3 days. The Rhizobium *Sinorhizobium* meliloti 1021 strain was used for inoculation. Rhizobium was cultured with TY (tryptone, yeast extract, and sodium chloride) medium (supplemented with 10 mg mL^–1^ tetracycline, 200 mg mL^–1^ streptomycin, and 6 mmol L^–1^ calcium chloride) in 28°C shaker till it reaches OD_600_ value of 1.0 (Wang et al., 2018). After that, 5 mL of rhizobial suspension diluted to OD_600_ = 0.1 was inoculated to the roots of 5 days seedlings. Nodule numbers were identified 21 days post inoculation (dpi). For time course assays, nodules of wild type were harvested at 7, 14, and 21 dpi.

### Nitrogenase Activity

Nitrogenase activity was measured by acetylene reduction assay (ARA) (Hardy et al., 1968). The roots with nodules of wild type, *mthan1-1 mthan2-1*, *35S:MtHAN1*, and *35S:MtHAN2* were introduced into 120 mL bottles sealed with rubber stoppers, and each bottle contained three plants. 12 mL of air was replaced with 12 mL of acetylene and the bottles were incubated at room temperature for 2 h. 1 mL gas in each bottle was used to measure the production of ethylene in a Shimadzu GC 2014C gas chromatograph using a porapak N column (Shimadzu, Kyoto, Japan).

### RNA Extraction, RT-PCR and Real-Time PCR Analysis

Total RNA of different tissues was extracted from 6-weeks-old plants, and RNA of nodules was extracted 3 weeks post inoculation with rhizobia. Total RNA was isolated using Trizol-RT Reagent (Molecular Research Center, INC). RT-PCR analysis was performed as described previously (Zhou et al., 2011). Real-time PCR was performed using the Fast Start Essential DNA Green Master mix (Roche) and the Bio-Rad CFX Connect TM sequence detection system was used for data acquisition and analysis. *MtUBIQUITIN* (*Medtr3g110110*) was used for normalization. The primers used for Real-Time PCR are listed in Supplementary Table 1 online.

### Protoplast Transient Assay

To construct the effector plasmids, the CDS of *MtHAN1* was cloned into pBI221 by LR interaction. For the reporter plasmids, the promoter fragments of *NCR* genes *Medtr1g042940*, *Medtr5g072205* and *Medtr5g072275* were cloned into the pGreenII-0800 vector, which contains the LUC reporter gene at C-terminus, using restriction sites *Pst* I and *Bam* H I. The reporter (2 μg) and effector plasmids (8 μg) were co-transfected into protoplasts isolated from rosette leaves of 3-weeks-old Col-0 plants by the PEG-mediated transfection method (Yoo et al., 2007). The LUC activity in each cell lysate was measured by the Luciferase Assay System Kit (Promega).

## Results

### Identification of the *HAN* Homologs in *M. truncatula*

**FIGURE 1:**
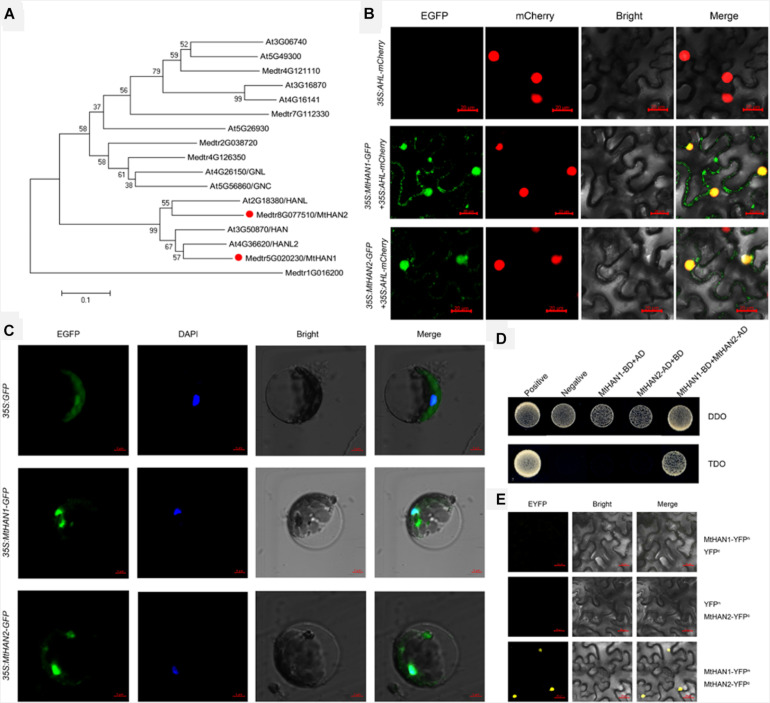
*Medicago truncatula HANABA TARANU* (*HAN*) homologues interact in nucleus. **(A)** Phylogenetic analysis of *HAN* genes among different species. **(B)** The subcellular localization of *MtHAN1*/*2*-GFP in *Nicotiana benthamiana* leaves. At-Hook Motif Containing Nuclear Localized (AHL)-mCherry was used as a marker for the nucleus. Bars = 20 μm. **(C)** The subcellular localization of *MtHAN1*/*2*-GFP in *M. truncatula* protoplasts. Free GFP was used as a control. Bars = 5 μm. **(D)** Physical interaction between *MtHAN1* and *MtHAN2* by yeast two-hybrid assay. Interaction was examined by yeast grown on TDO (SD-Leu-Trp-His) medium. **(E)** BiFC showing the interaction between *MtHAN1* and *MtHAN2* in nucleus. Bars = 20 μm.

To identify the homologs of *HAN* in *M. truncatula*, a BLASTP search using the Arabidopsis *HAN* against *M. truncatula* genome database was performed and two candidate genes, *Medtr5g020230* and *Medtr8g077510*, were obtained. Phylogenetic analysis and protein alignment showed that these two genes were close homologs of *HAN*, *HANL*, and *HANL2*, thus, were named *MtHAN1* and *MtHAN2* (Figure 1A and Supplementary Figure 1). Subcellular localization of *MtHAN1* and *MtHAN2* (*MtHANs*) revealed that they localized to the nucleus and cytoplasm. The At-Hook Motif Containing Nuclear Localized (AHL) protein was used as a control (Figure 1B). A similar localization of *MtHANs* was also observed in protoplasts of *M. truncatula* (Figure 1C). A yeast two-hybrid (Y2H) assay was performed to test whether *MtHAN1* and *MtHAN2* can physically interact (Figure 1D). The results indicate that *MtHAN1* and *MtHAN2* do interact, and so presumably form a heterodimer as has been reported for *HAN* and its *HANL* homologs in Arabidopsis (Zhang et al., 2013). Moreover, in bimolecular fluorescence complementation (BiFC) assays *MtHAN1* and *MtHAN2* were found to only interact in the nucleus (Figure 1E), consistent with their predicted roles as transcription factors.

### Identification the Loss-of-Function Mutants of *MtHAN1* and *MtHAN2* in *M. truncatula*

**FIGURE 2:**
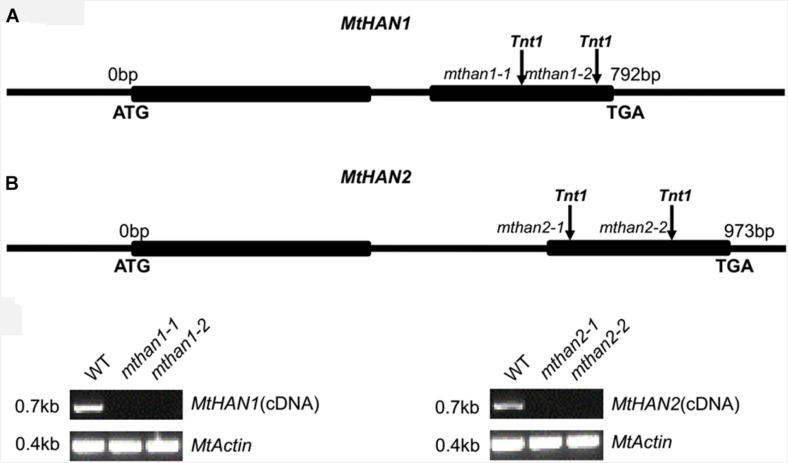
Molecular characterization of *MtHANs* in *M. truncatula*. **(A)** Schematic diagram of the gene structure of *MtHAN1* and *MtHAN2*. Vertical arrows mark the positions of the *Tnt1* insertions. Boxes represent exons and lines represent introns and UTRs. **(B)** RT-PCR analysis of *MtHAN1* and *MtHAN2* transcripts in the wild type (WT) and mutant alleles. *MtActin* was used as a loading control. 28 cycles were used for the RT-PCR.

To explore the functions of *MtHAN1* and *MtHAN2*, a reverse genetic screening was performed in a *Tnt1* retrotransposon-tagged mutant collection of *M. truncatula* (Cheng et al., 2014). Two independent *Tnt1* insertion mutants, all with insertions in exon 2, for both *MtHAN1* (*mthan1-1* and *mthan1-2*) and *MtHAN2* (*mthan2-1* and *mthan2-2*), were isolated (Figure 2A). Amplification of full-length cDNA showed that the transcripts of *MtHAN1* and *MtHAN2* were abolished in their respective mutants (Figure 2B). These data indicate that all four alleles are knockout mutants. Phenotypic observation showed that there are no obvious developmental defects in the leaves or flowers of single mutants (Supplementary Figure 2). To assess functional redundancy between *MtHAN1* and *MtHAN2*, double mutants were generated. However, the plants from two mutant combinations (*mthan1-1 mthan2-1* and *mthan1-2 mthan2-2*) grew normally, and did not show any visible defects in leaf and flower development at the vegetative and reproductive stages (Supplementary Figure 2). These results imply that *MtHANs* may play different roles in *M. truncatula*, compared with those of *HAN* in Arabidopsis.

### *MtHAN1* and *MtHAN2* Play a Redundant Role in Root Development

**FIGURE 3:**
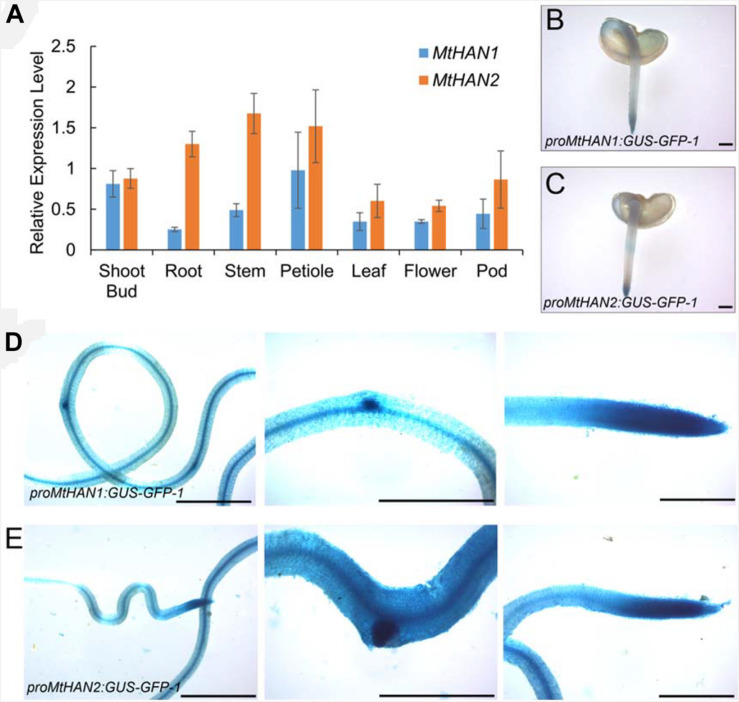
Expression patterns of *MtHAN1* and *MtHAN2*. **(A)** The expression patterns of *MtHAN1* and *MtHAN2* in tissues of shoot bud, root, stem, petiole, leaf, flower, and pod. **(B–E)** GUS expression in roots that were transformed with either *proMtHAN1:GUS–GFP* in panel **(B,D)** or *proMtHAN2:GUS–GFP* in panel **(C,E)**. **(B,C)** GUS staining in 5-days-old germinating seeds. **(D,E)** GUS staining in root vascular, lateral root primordial, and main root tip of 7-days-old root. Bars = 1 mm.

**FIGURE 4:**
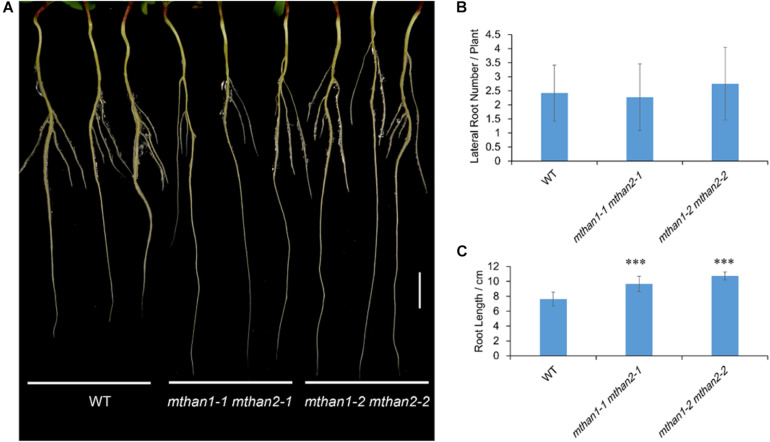
*MtHAN1* and *MtHAN2* are redundantly involved in the root elongation. **(A)** Seedling images of WT and *mthan1 mthan2* double mutant plants on the seventh day after transferring to MS plates. Three representative seedlings of each plant line were photographed. Bar = 1 cm. **(B)** Measurement of lateral root number. **(C)** Measurement of primary root length. Values are the means ± SD (*n* = 12). ^∗∗∗^*P* < 0.001.

To investigate the potential function of *MtHAN1* and *MtHAN2* in *M. truncatula* development, their expression patterns were analyzed. Tissue-expression analysis by qRT-PCR showed that both *MtHAN1* and *MtHAN2* were relatively highly expressed in the shoot bud, stem and petiole, with *MtHAN2* also showing high expression in the root (Figure 3A). To more comprehensively determine the expression patterns of *MtHAN1* and *MtHAN2*, promoter-GUS constructs were stably introduced into wild type, and GUS activity was examined in transgenic plants. The results showed that *MtHAN1* and *MtHAN2* displayed similar expression patterns in independent transgenic lines. GUS signals were detected in root tip of germinating seeds, in root vascular tissues, lateral root primordia, and primary root tips. No GUS staining was observed in the control (Figures 3B–E and Supplementary Figure 3). To assess if *MtHAN1* and *MtHAN2* function in root development, the root length and lateral root number were measured in wild type and mutants. The results showed that the lateral root number in the double mutants was similar to wild type, but the root length was increased in double mutants (Figure 4). These observations suggest that *MtHAN1* and *MtHAN2* are redundantly involved in the root elongation.

### *MtHAN1* and *MtHAN2* Are Involved in Nodule Formation

**FIGURE 5:**
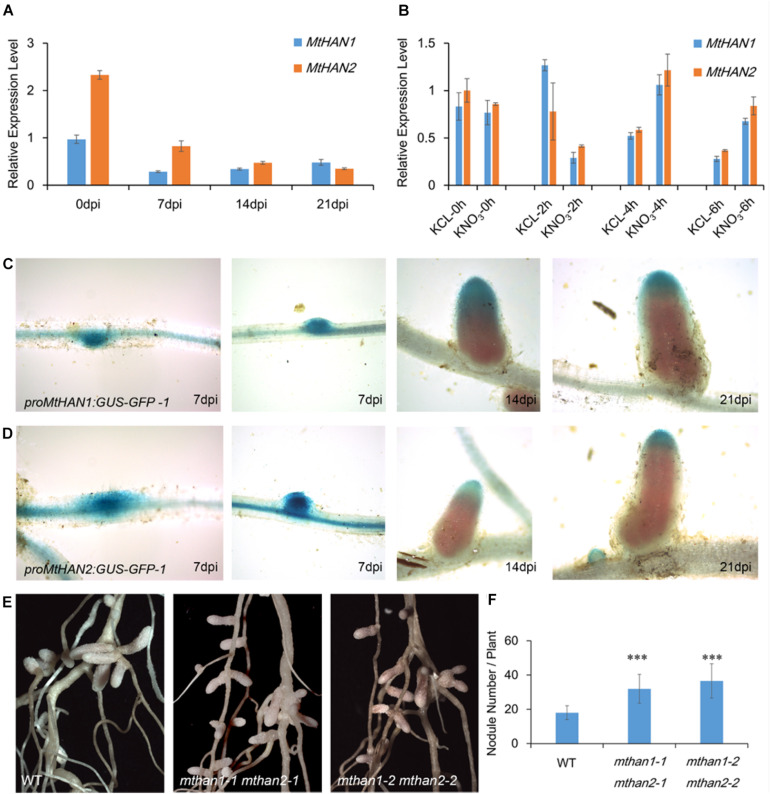
*MtHAN1* and *MtHAN2* express in nodules and the *mthan1 mthan2* double mutant has an increased nodule number. **(A)** Expression pattern of *MtHANs* in different developmental stages of nodule. The root (0 dpi) was used as control. Values are the means ± SD of three biological replicates. **(B)** Expression pattern of *MtHANs* in leaves and petioles after nitrate treatment, KCl treatment as control. Values are the means ± SD of three biological replicates. **(C,D)** *ProMtHAN1: GUS–GFP* and *proMtHAN2:GUS–GFP* reporter activity associated with the progressive stages in nodule development. **(E)** Photos of nodules from WT and *mthan1 mthan2* at 21 dpi. **(F)** The average number of nodules in WT and *mthan1 mthan2* plants. Values are the means ± SD (*n* = 16). ^∗∗∗^*P* < 0.001.

To investigate whether *MtHANs* play roles in nodule development, the expression pattern of *MtHANs* in different organs during nodulation was analyzed. qRT-PCR data showed that both *MtHAN1* and *MtHAN2* were mainly expressed in the shoot bud, stem and root, but were expressed at lower levels in the nodule (Supplementary Figure 4A). These results are consistent with the data of *MtHAN2* (the probeset of *MtHAN1* is not available) derived from *Medicago truncatula* Gene Expression Atlas (MtGEA) (Supplementary Figure 4B). In developing nodules, the expression level of *MtHAN1* was slightly increased, while the expression of *MtHAN2* was decreased (Figure 5A). Moreover, we also detected the expression of *MtHANs* in young nodules and mature nodules at 21 dpi. The results showed that the transcriptional level of *MtHAN1* was higher than that of *MtHAN2* in both kinds of nodules (Supplementary Figure 4C). A previous report showed that expression of the GATA factor *GATA, nitrate-inducible, carbon metabolism-involved* (*GNC*) is nitrate inducible in Arabidopsis (Bi et al., 2005). Nitrate concentration is critical for nodule formation. High levels of nitrate are able to inhibit multiple aspects of nodule development, including flavonoid production, rhizobial infection, nodule initiation, nodule growth, and nitrogen fixation activity. In addition, high nitrate can accelerate nodule senescence or disintegration (Nishida and Suzaki, 2018). To test whether *MtHANs* are able to respond to nitrogen, their expression levels were measured after treatment with KNO_3_, using KCl as a control treatment. The results showed that the expressions levels of *MtHAN1* and *MtHAN2* were reduced after nitrate application for 2 h, and then, increased after 4 and 6 h treatment (Figure 5B).

To further investigate their roles in nodulation, the expression patterns of *MtHANs* was analyzed in nodules. The *proMtHAN1:GUS* and *proMtHAN2:GUS* transgenic plants were inoculated with *S. meliloti* strain 1021. At 7 dpi, GUS signals were observed in the developing nodule primordia and young nodule. At 14 and 21 dpi, *MtHAN1* and *MtHAN2* expression was associated predominantly with the meristem and infection zone of nodules. No GUS staining was observed in the control (Figures 5C,D and Supplementary Figure 3). We also confirmed this result by the cross-section of roots and nodules after staining (Supplementary Figure 5). These data indicate that *MtHAN1* and *MtHAN2* are correlated with nodule development. It is noted that *MtHAN2* was expressed in the meristem as early as 14 dpi, therefore, the decreased expression observed for *MtHAN2* at later stages of nodule development was possible due to the growing fixation zone.

To further assess the roles of *MtHANs* during nodulation, wild type and mutants plants were inoculated with *S. meliloti* 1021 (Supplementary Figure 6). We then measured the nodule number in *mthan1-1* and *mthan2-1*. The result showed that the number of nodules in *mthan1-1* was similar with that of wild type, while the number of nodules in *mthan2-1* was slightly higher (Supplementary Figure 7A). Considering potential functional redundancy of the *MtHANs*, nodulation of the double mutants was evaluated. The nodule development was normal in *mthan1 mthan2* double mutants (Figure 5E). However, the number of nodules in the *mthan1 mthan2* double mutant was increased relative to wild type (Figure 5F). To assess whether there were any developmental defects in the *mthan1 mthan2* nodules, we made a longitudinal section of histochemically stained mature nodules at 21 dpi with *S. meliloti* 1021 carrying hemA/lacZ fusion. The result showed that the nodules of *mthan1 mthan2* had no obvious developmental defects (Supplementary Figure 8A,B). The measurement of nitrogenase activity also showed that nitrogenase activity of *mthan1-1 mthan2-1* was not altered compared with that of wild type (Supplementary Figure 8C). In addition, we measured the length of shoot and roots, and the biomass of nodulated wild type and double mutant plants. The results showed that the shoot length of double mutant was reduced compared with that of wild type (Supplementary Figure 9A). However, there was no difference in root length and biomass between wild type and mutants (Supplementary Figures 9B,C). Then, *MtHAN1* and *MtHAN2* were overexpressed in wild type plants. qRT-PCR data showed that the expression levels of *MtHANs* were significantly increased in the transgenic plants, and overexpression of *MtHANs* didn’t influence leaf, flower and root development (Supplementary Figure 10). Furthermore, we analyzed the number of nodules in wild type, *35S:MtHAN1* and *35S:MtHAN2* plants at 21 dpi. The results showed no difference in the number of nodules between wild type and *35S:MtHANs* plants (Supplementary Figure 11). Further analysis showed that overexpression of *MtHAN1* or *MtHAN2* didn’t affect nodule nitrogenase activity (Supplementary Figure 8C). These results indicate that *MtHANs* function in the repression of nodule formation.

### Transcriptomic Profiles of Nodules in *mthan1 mthan2*

**FIGURE 6:**
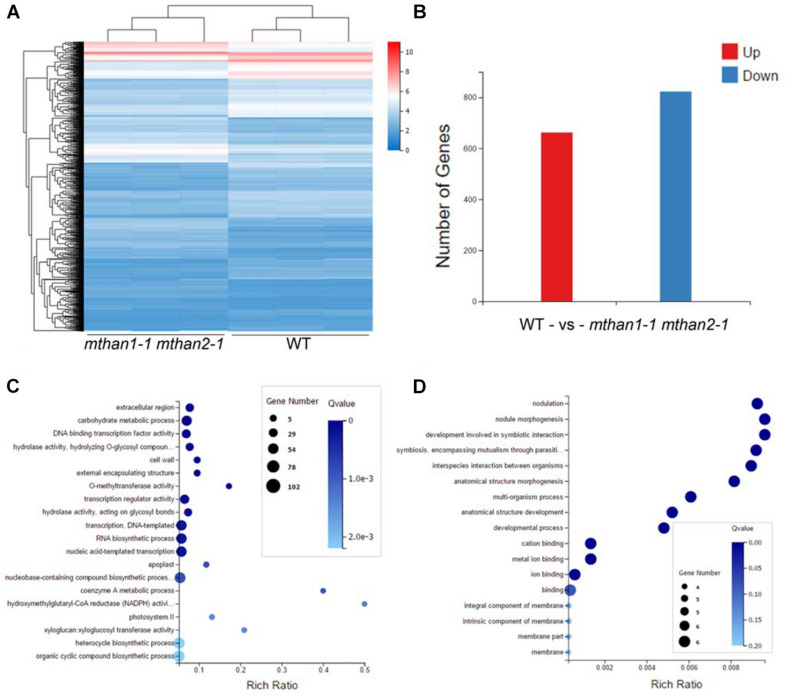
Different expression genes (DEGs) in *mthan1-1 mthan2-1* nodules and gene ontology (GO) enrichment of DEGs. **(A)** The heatmap of fragments per kilobase of transcript per million reads mapped (FPKM) values of 1,485 differential expressed genes in three biological replicates of nodules from WT and *mthan1-1 mthan2-1* plants. **(B)** The number of up-regulated and down-regulated genes in the *mthan1-1 mthan2-1* nodules at 21 dpi. **(C)** The top 20 significantly enriched GO term of DEGs. **(D)** GO enrichment associated to nodulation and symbiosis.

To understand the involvement of *MtHANs* in nodulation, we performed RNA-seq transcriptomic analysis using nodules from wild type and *mthan1-1 mthan2-1* 3 weeks after inoculation with *S. meliloti* 1021. Genes with more than twofold expression changes and the *P*-values less than 0.001 were identified as different expression genes (DEGs). In total, 1,485 DEGs were found significantly changed between wild type and *mthan1 mthan2*. Compared to wild type, 662 and 823 were up- and down-regulated, respectively, in *mthan1-1 mthan2-1* double mutant (Figures 6A,B and Supplementary Table 2). Multiple DEGs are involved in processes that are important for nodulation. Nod factor perception leads to root hair swelling and branching, which requires cell wall relaxation followed by a redirection of cell wall material secretion (Xie et al., 2012). Gene ontology (GO) terms enrichment analysis showed that, among which, the most enriched GO terms were extracellular region (GO:0005576), carbohydrate metabolic process (GO:0005975), DNA binding transcription factor activity (GO:0003700), hydrolase activity, hydrolyzing *O*-glycosyl compounds (GO:0004553), cell wall (GO:0005618), external encapsulating structure (GO:0030312) and *O*-methyltransferase activity (GO:0008171) (Figure 6C). Notably, that among DEGs having nodulation (GO:0009877) as a GO term. Nodule morphogenesis (GO:0009878) and development involved in symbiotic interaction (GO:0044111) was also enriched (Figure 6D). Flavonoid biosynthesis and phytohormones play important roles in legume-rhizobia recognition or nodule initiation and development (Murray et al., 2007; Suzaki et al., 2012; Roy et al., 2017; Gifford et al., 2018). Based on KEGG analysis, several secondary metabolite biosynthesis pathways were identified, including flavonoid biosynthesis (ko00941), monoterpenoid biosynthesis (ko00902), phenylpropanoid biosynthesis (ko00940), stilbenoid, diarylheptanoid, and gingerol biosynthesis (ko000945) and plant hormone signal transduction (ko04075) (Table 1 and Supplementary Figures 12–16). Examination of several of the major nodulation genes in the double mutant showed that their expression was essentially unchanged (Supplementary Table 3). These results implied that *MtHANs* affect nodulation through multiple pathways.

**TABLE 1:** Enriched KEGG pathways in different expression genes (DEGs).

KEGG_Term_ID	KEGG_Term	*q*-value
ko04712	Circadian rhythm–plant	7.00E-12
ko00941	Flavonoid biosynthesis	2.83E-06
ko00902	Monoterpenoid biosynthesis	1.86E-05
ko00940	Phenylpropanoid biosynthesis	0.00046
ko00945	Stilbenoid, diarylheptanoid and gingerol biosynthesis	0.000515
ko04075	Plant hormone signal transduction	0.000515
ko00196	Photosynthesis–antenna proteins	0.000943

### The *NCR* Genes Were Under the Regulation of *MtHANs*

**FIGURE 7:**
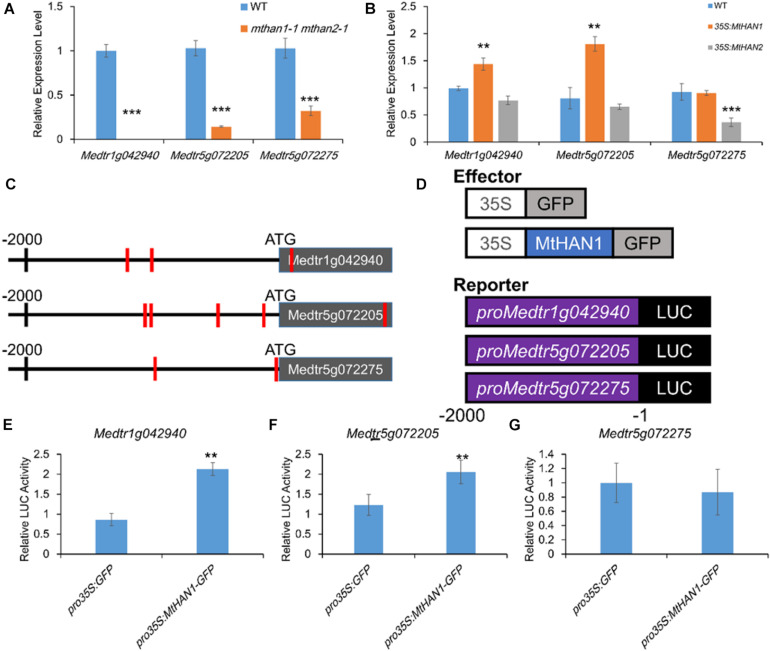
*MtHAN1* and *MtHAN2* regulate the expression of three *NCR* genes (gene models *Medtr1g042940*, *Medtr5g072205*, and *Medtr5g072275*) in nodules. **(A)** Relative expression of *NCRs* in nodules of *mthan1-1 mthan2-1* at 21 dpi. Values are the means ± SD of three biological replicates. ***P < 0.001. **(B)** Relative expression of *NCRs* in nodules of *35S:MtHAN1* and *35S:MtHAN2* at 21 dpi. Values are the means ± SD of three biological replicates. **P < 0.01. **(C)** Schematic diagram of the genes corresponding to *Medtr1g042940*, *Medtr5g072205*, and *Medtr5g072275*. Red vertical line indicates the *MtHANs*-binding motif (W-GATA-R). **(D)** Schematic structures of the effector and reporter constructs for the transient expression assay, in which *GFP* and *MtHAN1* were under the control of the *Cauliflower mosaic virus* (CaMV) 35S promoter, the firefly luciferase (*LUC*) reporter gene was driven by *NCR* promoters (*Medtr1g042940*, *Medtr5g072205*, and *Medtr5g072275*), and the Renilla luciferase (*LUC*; from *Renilla reniformis*) gene driven by the 35S promoter was used as an internal reference. **(E–G)** *MtHAN1* promotes the transcription of two *NCRs* (gene models *Medtr1g042940* and *Medtr5g072205*) in a protoplast transient assay. The GFP protein was used as negative control. Mean and SD values were obtained from three biological replicates. **P < 0.01, ***P < 0.001.

We noticed that amongst the *mthan1 mthan2* DEGs were three *NCR* genes, *Medtr1g042940*, *Medtr5g072205*, and *Medtr5g072275*, that had significantly decreased expression in the mutant. This was confirmed using qRT-PCR (Figure 7A). Previous studies showed that chemically synthesized cationic NCR peptides have antibacterial activity *in vitro*, and can suppress bacterial cell division in nodules (Tiricz et al., 2013). In the expression pattern database^3^ (Roux et al., 2014), two of three NCRs could be identified. It showed that the gene corresponding to gene model *Medtr1g042940* was expressed in FIId, FIIp, IZ, and ZIII and while another corresponding to *Medtr5g072205* was expressed in FIId, which overlapped or more distal to the expression domains of *MtHANs* (Supplementary Figure 17). To investigate whether *MtHANs* are able to regulate the transcription of the three *NCR* genes, we first detected the expression level of the three *NCR* genes in 21 dpi nodules of *mthan1-1* and *mthan2-1*. The result showed that there was a decrease in the expression of the gene corresponding to *Medtr5g072205* and *Medtr5g072275* in *mthan1-1*, while there was a decrease in the expression of the genes corresponding to *Medtr1g042940* and *Medtr5g072205* in *mthan2-1* (Supplementary Figure 7B). These data suggest that *MtHAN1* and *MtHAN2* play the redundant roles in the regulation of expression of the NCR corresponding to *Medtr5g072205*, while *MtHAN1* and *MtHAN2*, respectively, regulate the expression of the genes corresponding to *Medtr5g072275* and *Medtr1g042940*. Then, the expression levels of the three *NCR* genes were measured in nodules of *35S:MtHAN1* and *35S:MtHAN2* transgenic plants. The result showed that transcripts related to *Medtr1g042940* and *Medtr5g072205* were up-regulated in nodules of *MtHAN1*-overexpressing plants, implying that *MtHAN1* may activate the expression of these two *NCR* genes (Figure 7B). To test whether *MtHAN1* is able to activate the expression of *NCR* genes, the promoter sequences of three *NCR* genes were analyzed. Multiple binding motif of GATA factors were found in these promoters (Figure 7C). Then, transient expression assays were performed. The luciferase reporter constructs driven by 2-kb promoters of the three *NCR* genes were co-transformed, respectively, with *MtHAN1* effector proteins into Arabidopsis protoplasts (Figure 7D). The results showed that luminescence intensity driven by the promoter of *Medtr1g042940* and *Medtr5g072205* was increased significantly, compared with the GFP control effector protein (Figures 7E–G). These results imply that *MtHAN1* is able to recognize the promoters of these two genes in protoplasts and activates their expression. Taken together, these finding indicates that the transcriptional regulation of *MtHANs* on *NCR* genes is very complicated. Although *MtHAN1* is able to activate the expression of *Medtr1g042940* and *Medtr5g072205*, the downregulation of these genes in the double mutant was partially dependent on the functional redundancy of *MtHAN1* and *MtHAN2*.

### *MtHAN1* and *MtHAN2* Regulate the Expression of Peptidase and Peptidase Inhibitor Genes in Nodules

In plant, peptidases participate in many developmental processes, and biotic and abiotic stresses by regulating protein function. To ensure proper levels of proteolysis, the peptidase activity is precisely controlled by peptidase inhibitors at the protein level (Santamaria et al., 2014). All NCR peptides have a conserved signal peptide, which is cleaved by the signal peptidase DNF1 before secretion (Van de Velde et al., 2010). In this study, transcriptome profile analysis revealed that in addition to the three *NCR* genes, the expression of many peptidase or peptidase inhibitors also changed significantly (Table 2), even though the function of these genes has not been well studied in nodule formation. This includes a small decrease in the expression of *DNF1*, which has been directly implicated in nodule function through its role in processes NCR peptides. Taken together, these results suggest that *MtHANs* may regulate nodule formation by influencing the expression and processing of small peptides, including NCRs.

**TABLE 2:** Peptidase and peptidase inhibitor genes differentially expressed in *mthan1.mthan2*.

Gene ID	Annotation	log_2_ (*mthan1 mthan2*/WT)	*Q*-value (WT-vs-*mthan1 mthan2*)	*P* value (WT-vs-*mthan1 mthan2*)
Medtr0268s0040	Peptidase	2.46	0.00089	0.0002414
Medtr1g025370	Peptidase	1.26	4.44E-08	6.72E-09
Medtr1g026380	Peptidase	2.69	1.09E-29	5.91E-31
Medtr1g040370	SCPL family	1.86	3.71E-38	1.66E-39
Medtr1g072420	Peptidase	−1.88	0	0
Medtr1g075340	Peptidase inhibitor	−1.30	2.22E-14	2.18E-15
Medtr1g079120	SBT1	−2.57	7.33E-06	1.43E-06
Medtr1g093100	Peptidase	−2.05	6.82E-63	2.01E-64
Medtr2g022460	Peptidase	1.22	2.85E-287	1.86E-289
Medtr2g022480	Peptidase	−1.51	7.68E-254	5.69E-256
Medtr2g022490	Peptidase	−4.70	0	0
Medtr2g027315	Peptidase	3.99	0.0005204	0.0001351
Medtr2g095750	SCPL family	−1.22	2.01E-37	9.15E-39
Medtr3g087440	FAR1	5.14	0.0002411	5.88E-05
Medtr3g096930	Peptidase	1.04	0.0008142	0.0002194
Medtr3g100500	Peptidase	−1.68	0	0
Medtr3g463590	Peptidase inhibitor	−2.14	2.38E-21	1.71E-22
Medtr4g047610	SAG family	−1.46	4.36E-27	2.55E-28
Medtr4g057570	SCPL family	−1.78	0.0003861	9.73E-05
Medtr4g057585	SCPL family	−1.28	2.79E-27	1.62E-28
Medtr4g077320	Peptidase	1.53	0.0002637	6.49E-05
Medtr4g079770	SAG family	7.46	7.90E-20	6.01E-21
Medtr4g089155	SCPL family	1.04	0	0
Medtr4g094918	RD21B	1.90	2.04E-23	1.34E-24
Medtr4g100990	Peptidase	−1.53	1.66E-22	1.14E-23
Medtr4g123995	SGO2	5.50	4.41E-06	8.32E-07
Medtr5g022560	SAG family	−1.48	0	0
Medtr5g080890	CDR1	2.57	7.58E-06	1.48E-06
Medtr6g078140	Peptidase inhibitor	−9.26	3.45E-56	1.10E-57
Medtr6g078250	Peptidase inhibitor	−5.62	1.23E-06	2.17E-07
Medtr6g078260	Peptidase inhibitor	−8.74	3.20E-83	7.21E-85
Medtr6g078280	Peptidase inhibitor	−8.73	0	0
Medtr6g078290	Peptidase inhibitor	−3.19	9.05E-54	2.98E-55
Medtr7g077340	Peptidase inhibitor	1.08	5.78E-81	1.34E-82
Medtr7g107100	AMP1	1.84	0	0
Medtr3g027890	DNF1	−0.19	4.84E-38	2.17E-39

## Discussion

GATA transcriptional factors were first identified because of their roles in the regulation of light responsive genes (Buzby et al., 1990; Gilmartin et al., 1990; Lam et al., 1990; Borello et al., 1993) and their ability to bind the GATA core sequence (Lowry and Atchley, 2000). Further studies showed that GATA factors play key roles in different organ development in Arabidopsis (Nishii et al., 2000; Shikata et al., 2004; Zhao et al., 2004; Liu et al., 2005; Nawy et al., 2010; Ding et al., 2015). In this study, the homologs of *HAN* and *HANL*, *MtHAN1* and *MtHAN2*, were identified in *M. truncatula*. Surprisingly, subcellular localization shows that both *MtHAN1* and *MtHAN2* are localized not only in the nucleus, but also in the cytoplasm. However, *MtHAN1* and *MtHAN2* were only able interact with each other in nucleus, implying that like *HAN* and *HANL* in Arabidopsis, they can form the heterodimer in nucleus (Zhang et al., 2013).

Previous reports show that *HAN* plays a role in flower development in Arabidopsis. Loss-of-function of *HAN* leads to decrease in numbers of petals and stamens and fused sepals (Zhao et al., 2004). In addition, three other GATA family genes, *HANL2*, *GNC*, and *GNL*, play redundant roles with *HANs* in regulating flower development (Zhang et al., 2013). In *M. truncatula*, however, *mthan1*, *mthan2* and their double mutant did not show any obvious defects in flowers. It is possible that *MtHANs* do not participate in flower development, or that the homologs of *GNC* and *GNL* may replace the functions of *MtHANs* in regulating flower development in *M. truncatula*. Either way, this finding suggests that the functional diversity of GATA factors between Arabidopsis and *M. truncatula*.

It was demonstrated that the expression of *GNC* is inducible by nitrate in Arabidopsis (Bi et al., 2005). In our experiments, the expression levels of *MtHANs* were initially suppressed and then later on increased after nitrate treatment in *M. truncatula*, further supporting a role for GATA factors in plant N-responses. Moreover, the expression patterns of *MtHAN1* and *MtHAN2* show that they are mainly expressed in nodule primordia and the infection zone of mature nodules, implying the involvement of *MtHANs* in nodulation. The nodule number was increased in the *mthan1 mthan2* double mutant, indicating that *MtHANs* are negative regulators of nodule formation. *NCR* genes, which have different expression patterns, play important roles in the regulation of nodule number in *M. truncatula*. *NCR247* is expressed in older cells of the proximal infection zone and in the interzone. It interacts with FtsZ in the rhizobia, which is required for septum formation, and is thought to inhibit bacterial cell division to promote differentiation of bacteroids in *M. truncatula* nodules (Farkas et al., 2014). Another NCR encoding gene, *NFS1*, is also expressed in the proximal infection zone and the interzone, and functions in provoking bacterial cell death and early nodule senescence (Yang et al., 2017). *NFS2* is predominantly expressed the interzone of functional (Fix^+^) nodules, where it promotes bacterial lysis after differentiation (Wang et al., 2017). These studies indicate that some *NCRs* are expressed in different nodule zones, and play different roles in nodulation in *M. truncatula*. In this study, the expression levels of three *NCR* genes are significantly decreased in nodules of *mthan1 mthan2*. Given that *NCRs* are expressed and function in mature nodules, it seems unlikely they are involved in the increased nodule numbers observed in the *mthan1 mthan2* double mutant. Nonetheless, the changed expression of DNF1, the signal peptidase that processes NCRs, and many other peptidase and peptidase inhibitors in *mthan1 mthan2*, suggests that this important nodule system is perturbed. On the other hand, global gene transcriptional analysis indicates that several other pathways are influenced, which are important for nodule development.

Nodule number in *Medicago* is regulated mainly by ethylene, which control infections, and AON, which systemically controls nodule number. AON operates through CLE peptides that are released in the root and travel to the shoot, where they are perceived by the SUNN receptor kinase resulting in inhibition of nodule formation (Mortier et al., 2010). In Arabidopsis *HANs* act synergistically with the *SUNN* homologue *CLAVATA*, also a CLE peptide receptor, to regulate the shoot meristem. One intriguing possibility is that the enhanced nodulation of *MtHANs* is due to interference with AON, an idea that can be investigated in the future.

In this study, we identify the homologs of *HAN* and *HANL* in *M. truncatula* and further characterize their mutants. We find that in contrast to the role of *HAN* in flower development in Arabidopsis, *MtHANs* negatively regulate nodule development. This work expands our knowledge of the functions of *MtHANs* in *plants*, and gives an intriguing lead for future studies of regulation of nodule number in legumes. Further molecular and genetic studies will shed light on the mechanisms how *MtHANs* regulate this important developmental process.

## Data Availability Statement

The datasets presented in this study can be found in online repositories. The names of the repository/repositories and accession number(s) can be found below: SRA, PRJNA673405.

## Author Contributions

YX, CZ, and LH designed research. YX and HFW performed most research and analyzed the data. ZL, LW, ZG, XZ, GY, and HLW contributed analytical tools and performed some nodule analysis experiments. YX, CZ, and LH wrote the manuscript. All authors contributed to the article and approved the submitted version.

## Conflict of Interest

The authors declare that the research was conducted in the absence of any commercial or financial relationships that could be construed as a potential conflict of interest.

## Footnotes

1 http://www.genome.jp/tools/clustalw/

2 http://www.megasoftware.net/

3 https://iant.toulouse.inra.fr/symbimics
