# Supplementary material for: Developmental Analysis of the GATA Factor HANABA TARANU Mutants in Medicago truncatula Reveals Their Roles in Nodule Formation
Source: Front Plant Sci. 2021 Apr 29;12:616776. doi: 10.3389/fpls.2021.616776 (PMC8118203; doi:10.3389/fpls.2021.616776)
Supplement: Supplementary file 1 [file Data_Sheet_1.docx]

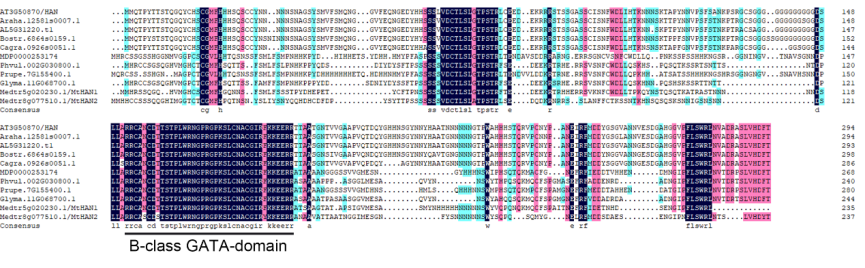


**FIGURE S1** Multiple protein sequence alignment of HAN and homologs from other species. Amino acids that are conserved throughout are shaded in different colors. The GATA-domain is indicated by a black line.


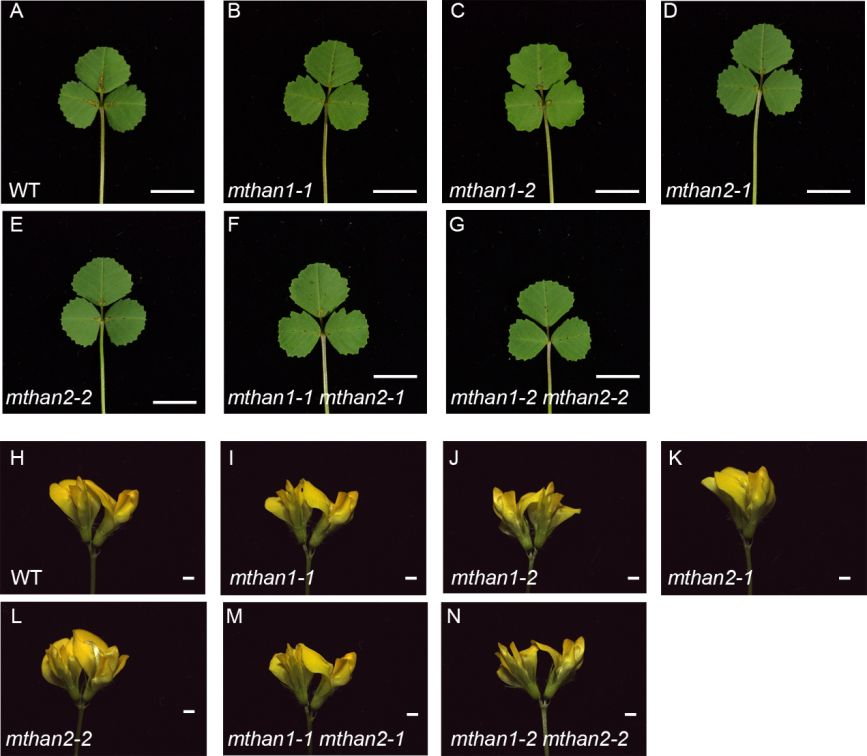


**FIGURE S2** Leaves and flowers of *mthan1*, *mthan2*, *mthan1 mthan2* mutants. (A-G) 4-week-old leaf of wild type (A), *mthan1-1* (B), *mthan1-2* (C), *mthan2-1* (D), *mthan2-2* (E), *mthan1-1* *mthan2-1* (F) and *mthan1-2* *mthan2-2* (G) plants. Bars=1cm. (H-N) 2-month-old flower of wild type (H), *mthan1-1* (I), *mthan1-2* (J), *mthan2-1* (K), *mthan2-2* (L), *mthan1-1* *mthan2-1* (M) and *mthan1-2* *mthan2-2* (N) plants. Bars=1mm.


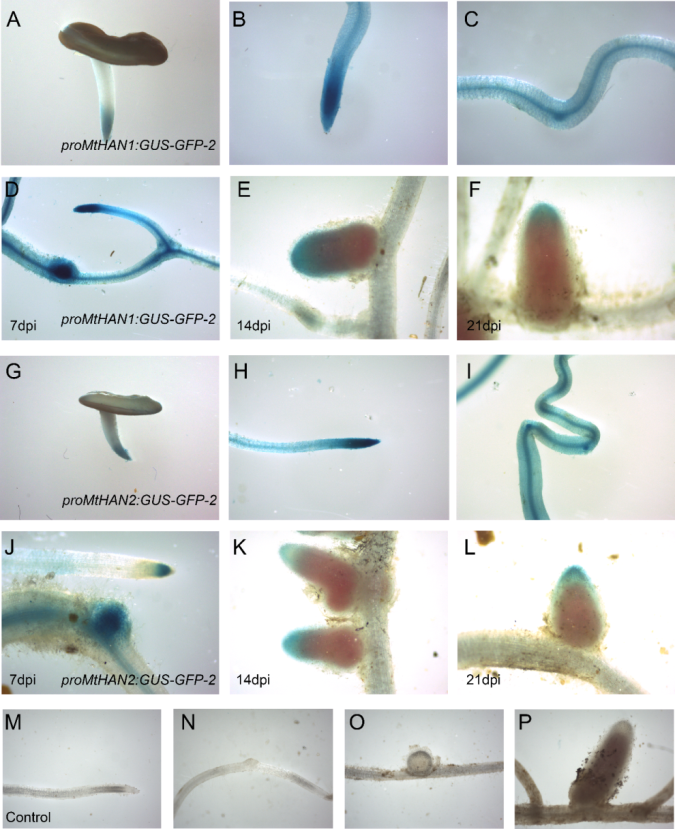


**FIGURE** **S3** Expression pattern of *MtHAN1* and *MtHAN2* in roots and nodules of a second independent transgenic line. (A-F) GUS staining of *proMtHAN1:GUS-GFP* transgenic plants in root tip of germinating seeds (A), primary root tip (B), lateral root primordia (C), young nodule at 7 dpi (D), nodule meristem at 14 dpi (E), nodule meristem and distal infection zone at 21 dpi (F). (G-L) GUS staining of *proMtHAN2:GUS-GFP* transgenic plants in root tip of germinating seeds (G), primary root tip (H), lateral root primordia (I), young nodule at 7 dpi (J), nodule meristem at 14 dpi (K), nodule meristem and distal infection zone at 21 dpi (L). (M-N) No GUS signals were detected in roots (M, N) and nodules (O, P) of wild type.


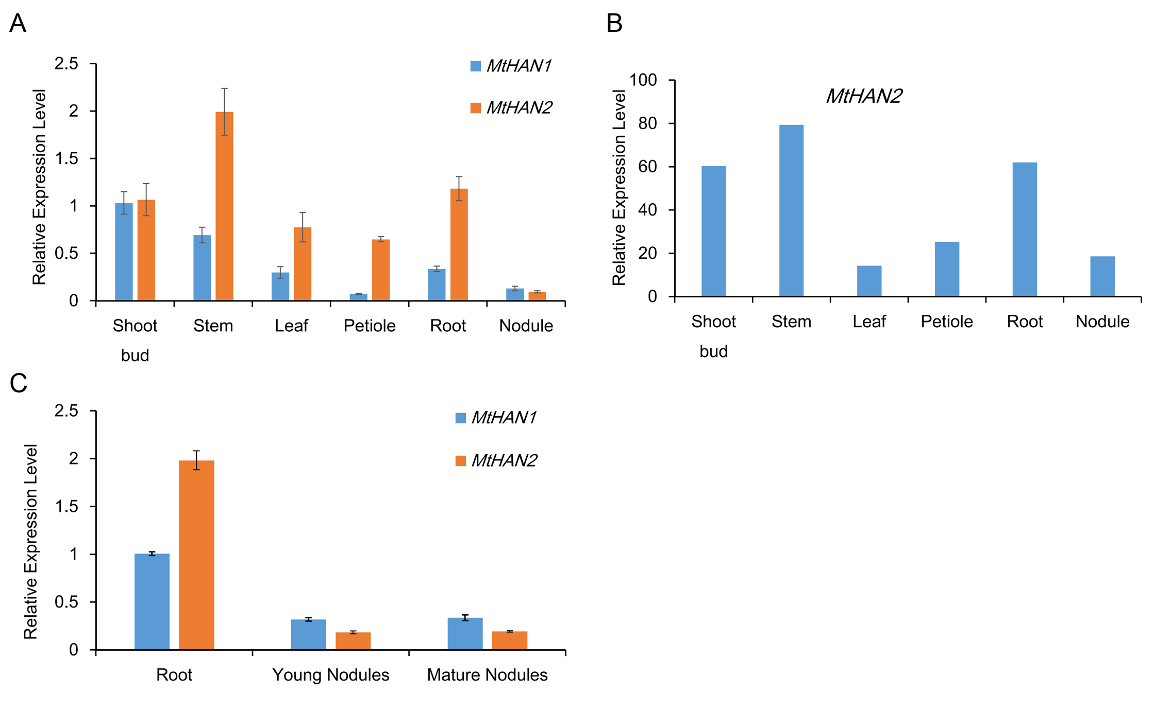


**FIGURE S4** Expression of *MtHANs* in different tissues. (A) The expression patterns of *MtHAN1* and *MtHAN2* in shoot bud, root, stem, petiole, leaf and nodule. (B) The expression profile of *MtHAN2* (Probeset, Mtr.27608.1.S1_s_at) in the *Medicago truncatula* Gene Expression Atlas (MtGEA). (C) The expression patterns of *MtHAN1* and *MtHAN2* in tissues of root, young nodules and mature nodules.


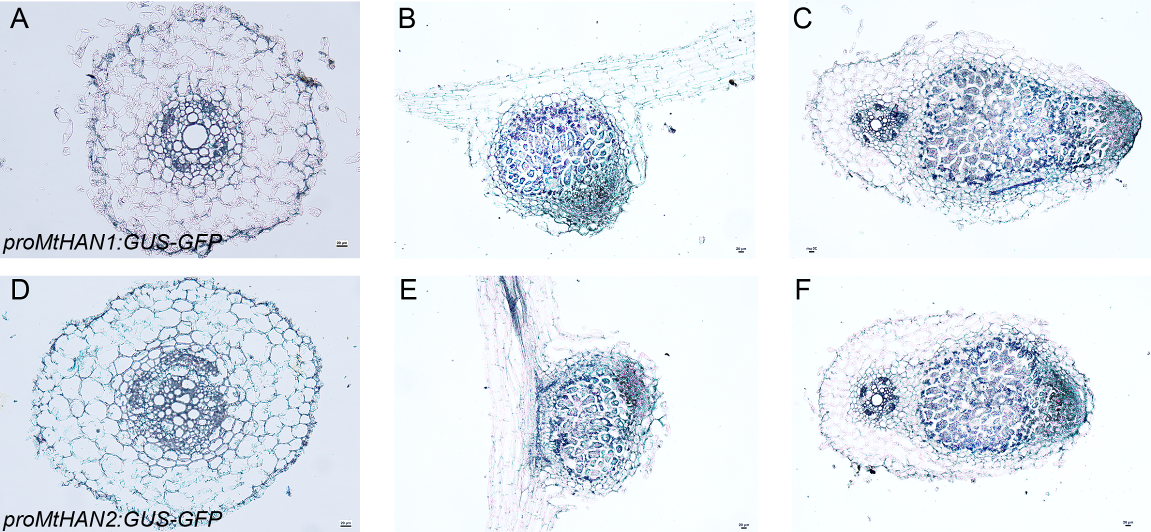


**FIGURE S5** Expression patterns of *MtHAN1* and *MtHAN2* in the sectioned roots and nodules after staining. (A-C) *proMtHAN1:GUS* signals were observed in the root vascular (A), young nodule (B), nodule meristem and infection zone in mature nodules (C). (D-F) *proMtHAN2:GUS* signals were observed in the root vascular (D), young nodule (E), nodule meristem and infection zone in mature nodules (F).


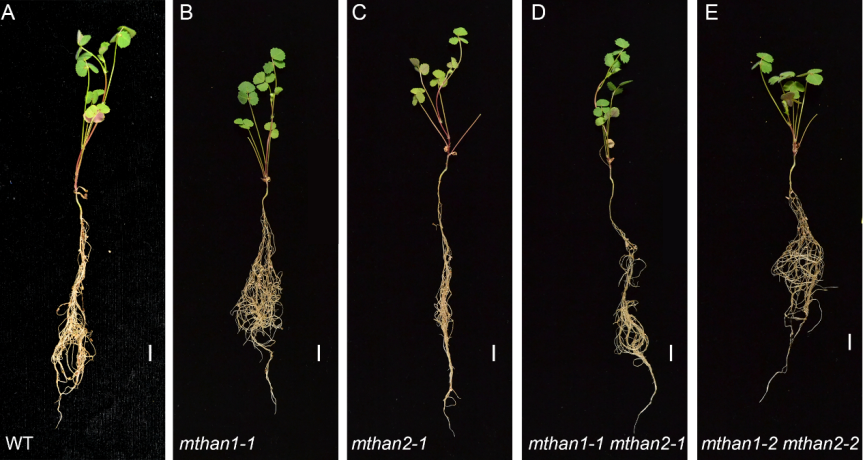


**FIGURE** **S6** Phenotype of seedlings after nodulation at 21 dpi with rhizobia. (A) Wild type. (B) mthan1-1. (C) mthan2-1. (D) mthan1-1 mthan2-1. (E) mthan1-2 mthan2-2. Bars=1cm.


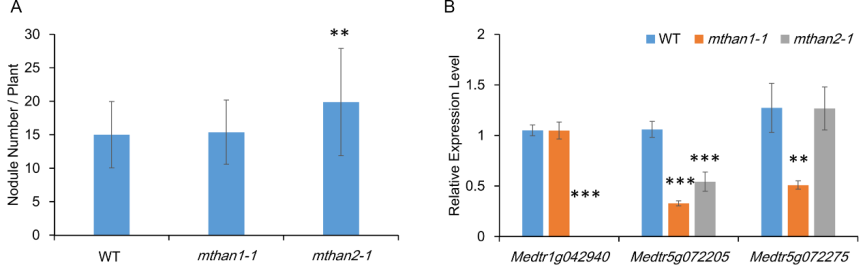


**FIGURE S7** The average number of nodules and expression level of three *NCR* genes in *mthan1-1* and *mthan2-1* single mutants. (A) The average number of nodules was increased in *mthan2-1* but didn’t change in *mthan1-1*. Values are the means ± SD (n=16). (B) The expression of three *NCR* genes in nodules of single mutants. Values are the means ± SD of three biological replicates. **P<0.01, ***P<0.001.


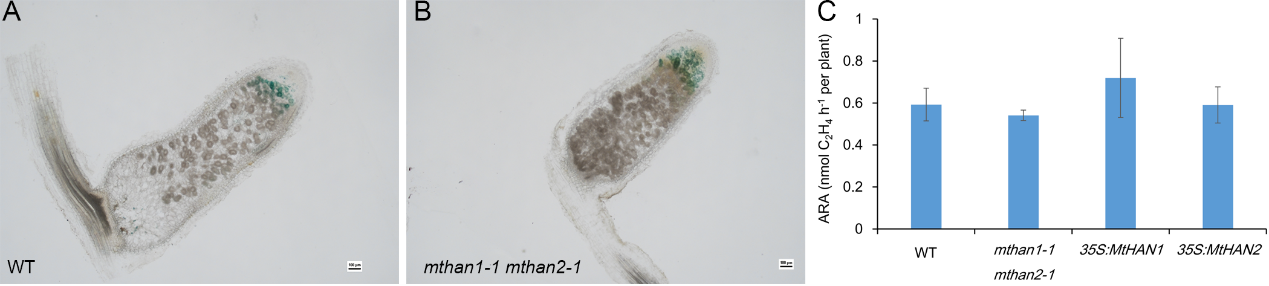


**FIGURE S8** *MtHANs* mutation did not impair nodule development and nitrogenase activity. (A-B) Longitudinal sections of wild type (A) and *mthan1-1 mthan2-1* (B) nodules stained by X-gal. Bars=100μm. (C) Nitrogenase activity of nodules at 28 dpi. Ethylene production was measured by acetylene reduction assay (ARA) in nodules. Values are the means ± SD of three biological replicates.


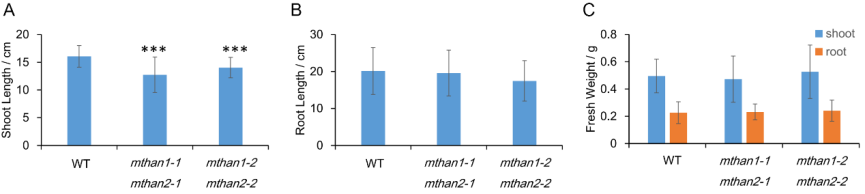


**FIGURE S9** The length of shoot and root, and biomass of *mthan1 mthan2* double mutant in nodulation. (A) The shoot length was reduced in *mthan1 mthan2* after nodulation. (B, C) the root length (B) and biomass (fresh weight) of shoot and root (C) didn’t change in *mthan1 mthan2* compared with those of wild type (WT) in nodulation. Values are the means ± SD (n=21). ***P<0.001.


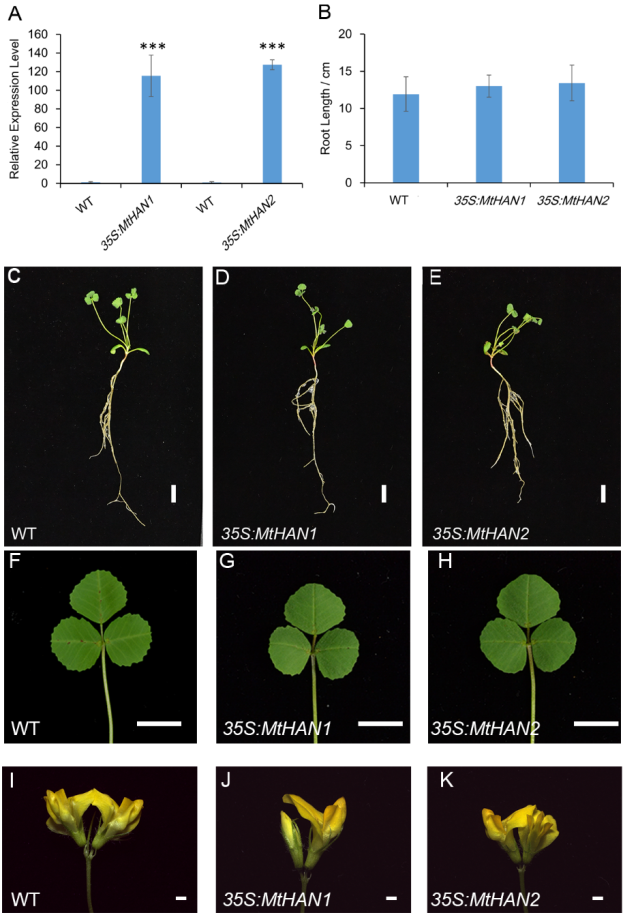


**FIGURE** **S10** Phenotype of *35S:MtHAN1* and *35S:MtHAN2* plants. (A) The relative expression of *MtHAN1* and *MtHAN2*. Values are the means ± SD of three biological replicates. ***P<0.001. (B) The root length of *35S:MtHAN1* and *35S:MtHAN2* plants. Values are the means ± SD (n=7). (C-E) The 7-day-old seedlings of wild type (C), *35S:MtHAN1* (D) and *35S:MtHAN2* (E) plants. Bars=1cm. (F-H) Leaves of 4-week-old wild type (F), *35S: MtHAN1* (G) and *35S:MtHAN2* (H) plants. Bars=1cm. (I-K) Flowers of 2-month-old wild type (I), *35S: MtHAN1* (J) and *35S: MtHAN2* (K) plants. Bars=1mm.


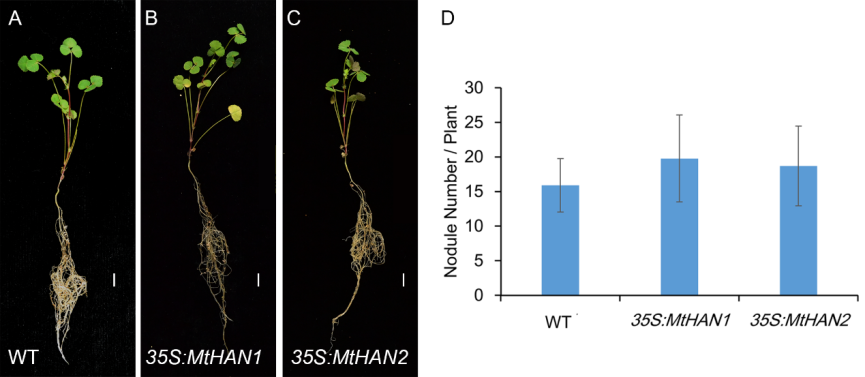


**FIGURE** **S11** Phenotype of seedlings after nodulation at 21 dpi. (A) Wild type. (B) 35S:MtHAN1. (C) 35S:MtHAN2. Bars=1cm. (D) The average nodule number of *35S:MtHAN1* and *35S:MtHAN2* plants. Values are the means ± SD (n=10).


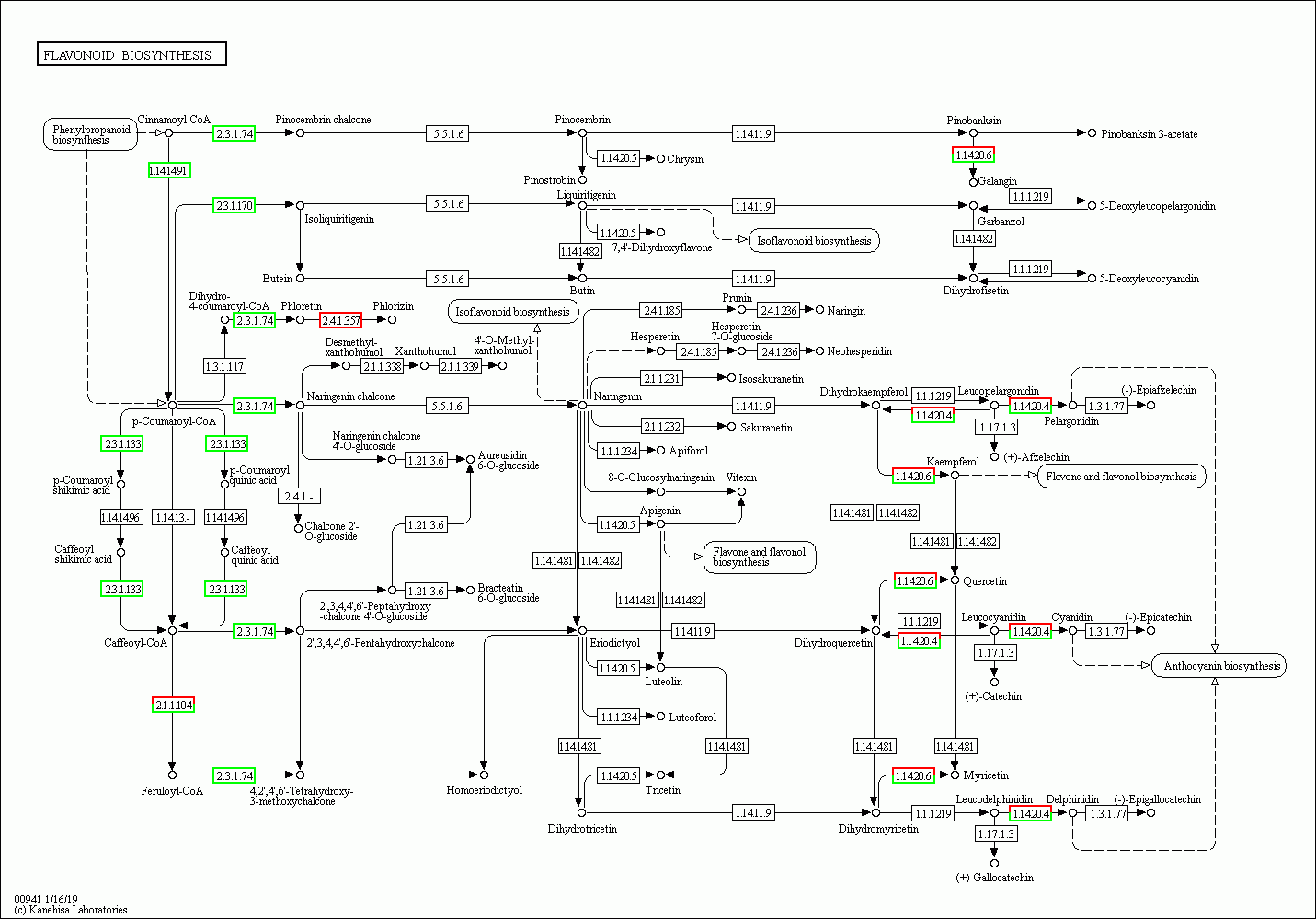


**FIGURE** **S12** The flavonoid biosynthesis pathway with induced and suppressed genes indicated in red and green frames, respectively.


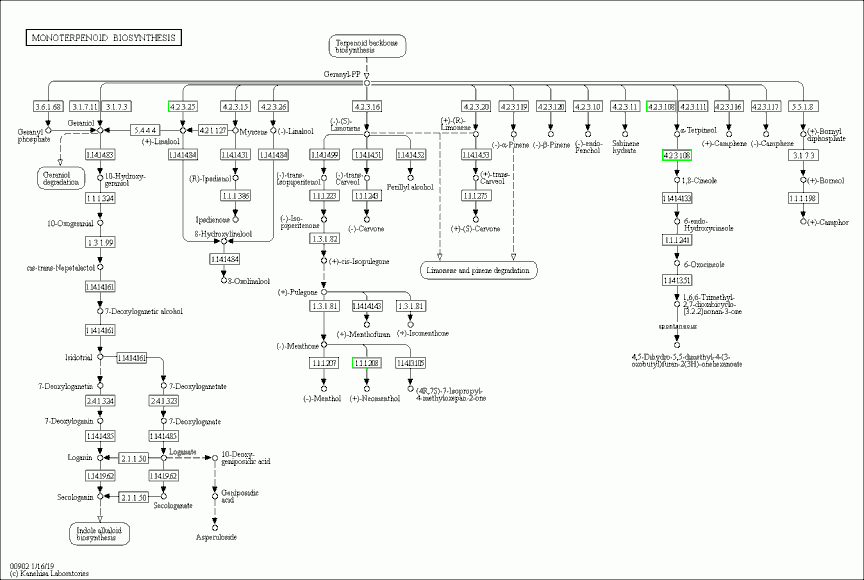


**FIGURE** **S13** The monoterpenoid biosynthesis pathway with induced and suppressed genes indicated in red and green frames, respectively.


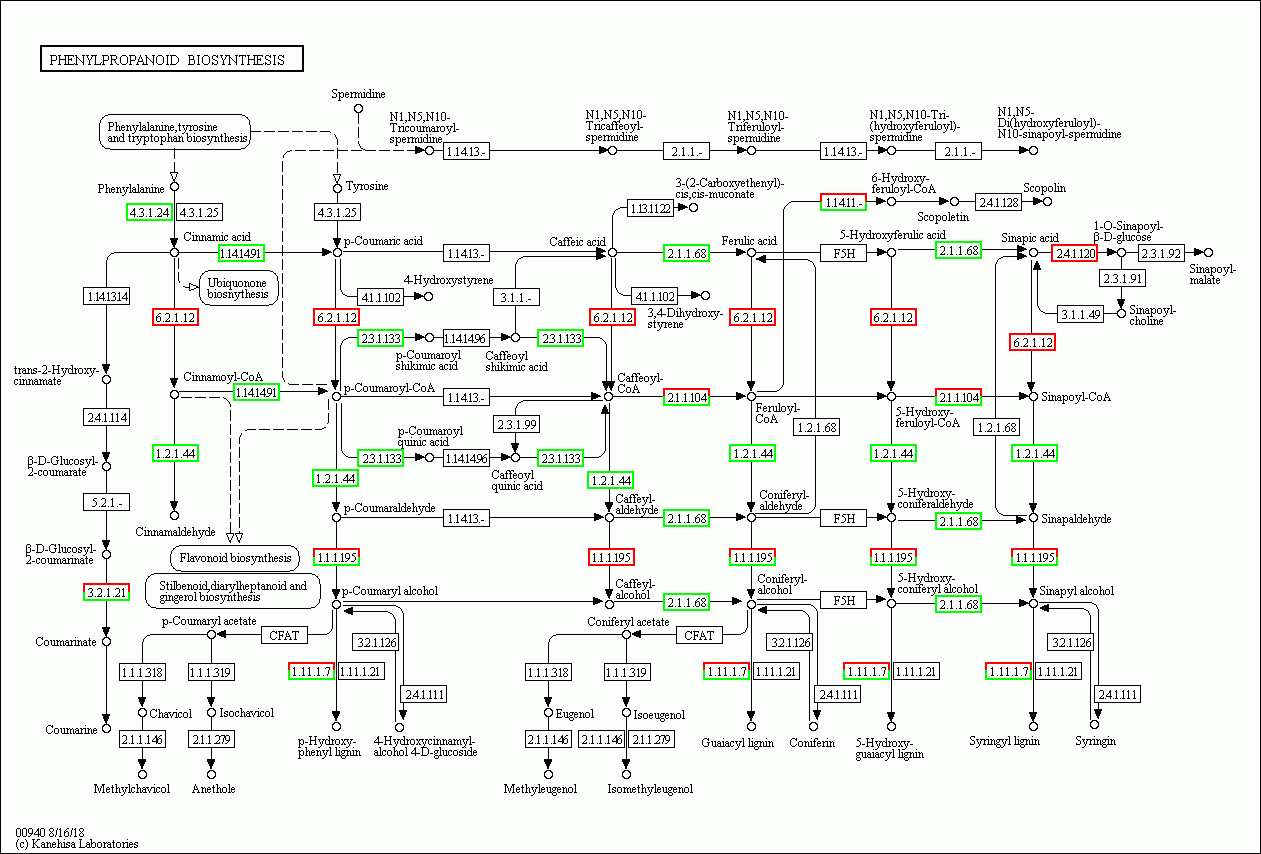
 **FIGURE** **S14** The phenylpropanoid biosynthesis pathway with induced and suppressed genes indicated in red and green frames, respectively.


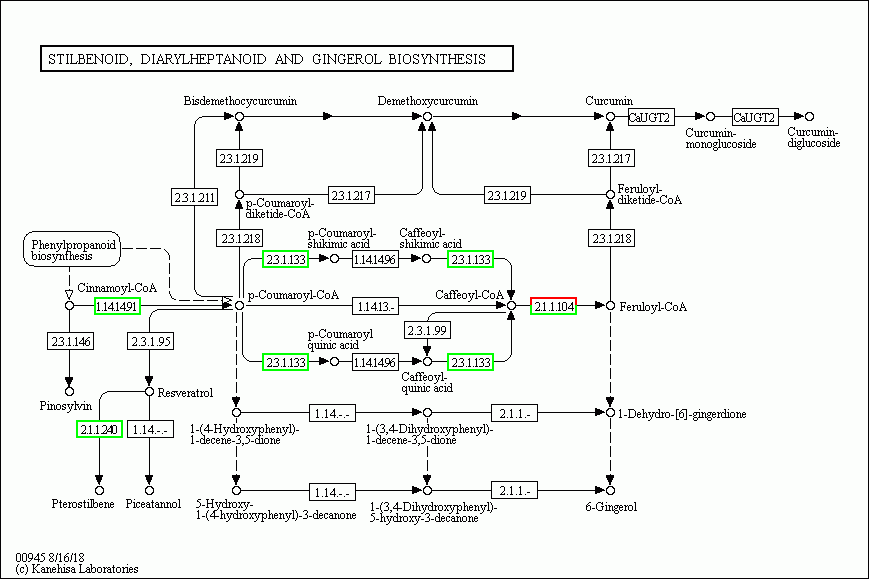


**FIGURE** **S15** Stilbenoid, diarylheptanoid and gingerol biosynthesis pathway with induced and suppressed genes indicated in red and green frames, respectively.


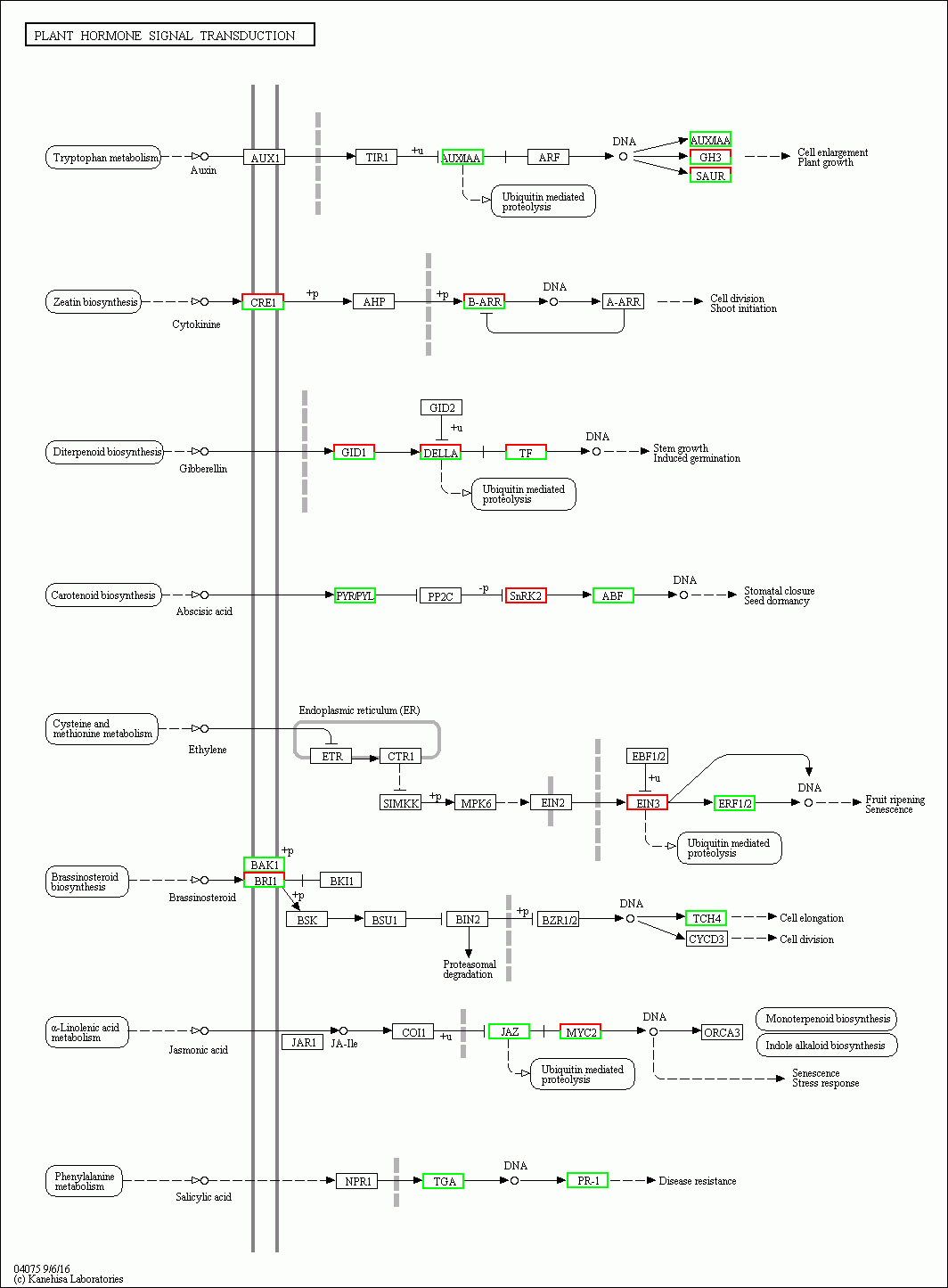


**FIGURE** **S16** Plant hormone signal transduction pathway with induced and suppressed genes indicated in red and green frames, respectively.


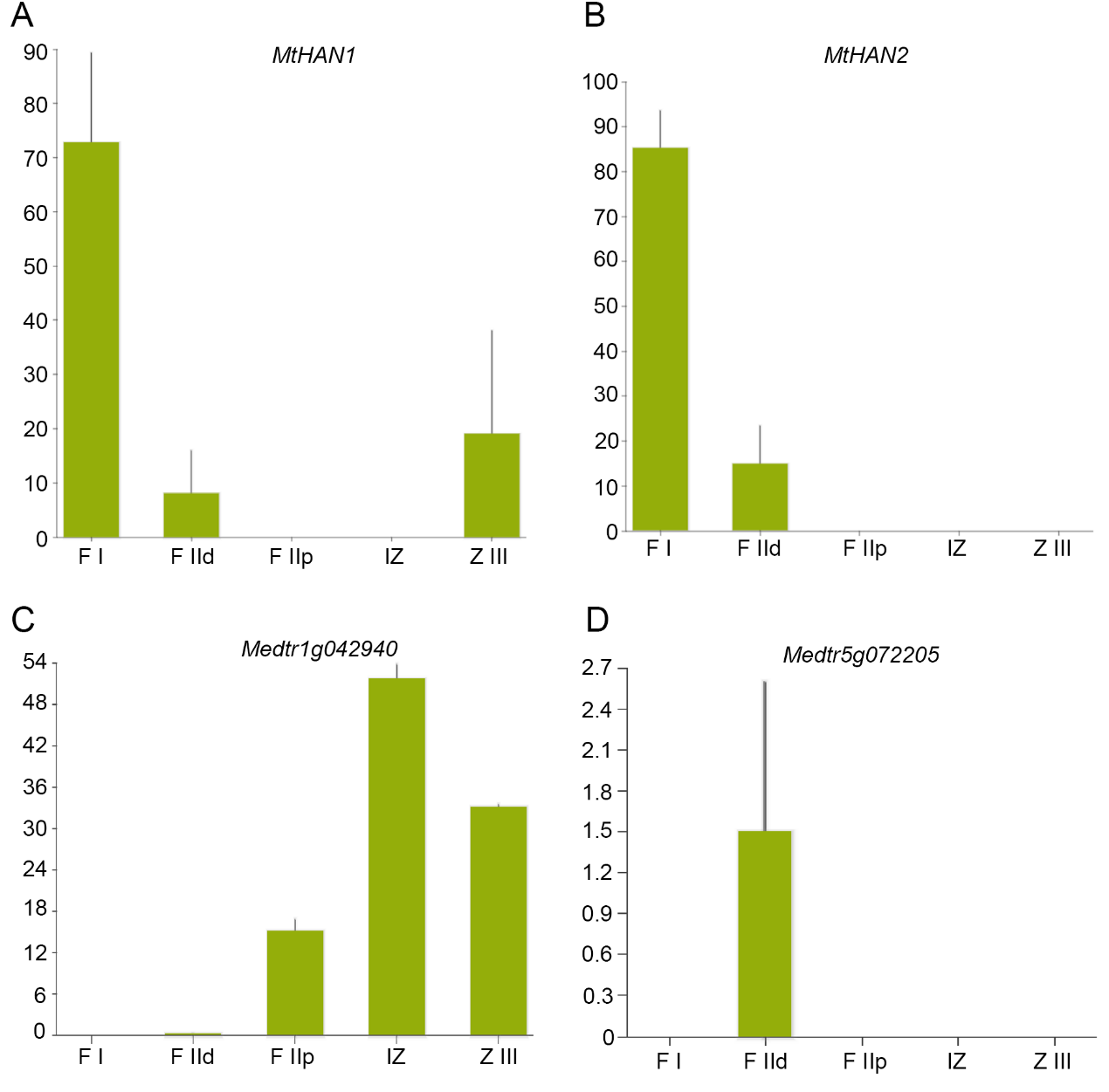


**FIGURE S17** The expression pattern of *MtHAN1*, *MtHAN2* and *NCR* genes in different nodule zones. (A) *MtHAN1.* (B) *MtHAN2*. (C) *Medtr1g042940.* (D) *Medtr5g072205*. FⅠ, fraction Ⅰ; FⅡd, distal fraction Ⅱ; FⅡp, proximal fraction II; IZ, interzone; ZⅢ, zone Ⅲ.
